# Different eGFR markers and prediction of cardiovascular risk

**DOI:** 10.1111/joim.70050

**Published:** 2025-11-22

**Authors:** Maria Tydén, Gorav Batra, Bengt Fellström, Claes Held, Johan Lindbäck, Inga Soveri, Maria K. Svensson, Ralph Stewart, Harvey D. White, Lars Wallentin

**Affiliations:** ^1^ Department of Medical Sciences Renal Medicine Uppsala University Uppsala Sweden; ^2^ Department of Medical Sciences Cardiology Uppsala University Uppsala Sweden; ^3^ Uppsala Clinical Research Center (UCR) Uppsala University Uppsala Sweden; ^4^ Green Lane Cardiovascular Service Auckland City Hospital and Auckland University Auckland New Zealand

**Keywords:** Keywords, cardiovascular events, creatinine, cystatin C, glomerular filtration rate, mortality

## Abstract

**Background:**

Renal dysfunction increases cardiovascular (CV) risk. We compared cystatin C‐based estimated glomerular filtration rate (eGFRcys), creatinine‐based eGFR (eGFRcr), and their ratio (eGFRcys/eGFRcr) in relation to major adverse cardiovascular events (MACE) and all‐cause mortality in chronic coronary syndrome, assessing the added prognostic value of the eGFRratio.

**Methods:**

In this post hoc analysis of 14,513 Stabilization of Atherosclerotic Plaque by Initiation of Darapladib Therapy trial patients, we investigated associations between baseline eGFRcys, eGFRcr, their ratio, and MACE and all‐cause death using Cox regression models, unadjusted and adjusted for eGFRcys, eGFRcr, and their combination. Discrimination was assessed using Harrell's *C*‐index; added value by the fraction of new information (FNI).

**Results:**

Median age was 65 years; 82% were male. Median eGFRcys was 77 (interquartile range [IQR]: 61–94) and eGFRcr 79 (IQR: 65–91) mL/min/1.73 m^2^. Over 3.7 years, 1449 MACE and 1063 deaths occurred. Lower eGFR values and eGFRratio were associated with increased MACE risk, primarily driven by CV death. For eGFRcys 60 versus 90, the hazard ratio (HR) for MACE adjusted for eGFRcr was 1.77 (95% CI: 1.49–2.09, FNI 54%). In contrast, eGFRcr adjusted for eGFRcys showed no positive association (HR 0.82, 95% CI: 0.68–0.97, FNI 3%). A lower eGFRratio was linked to higher MACE risk (HR 1.99, 95% CI: 1.80–2.21), which remained after eGFRcr adjustment (HR 1.89, 95% CI: 1.70–2.10, FNI 54%) but was attenuated after eGFRcys adjustment (HR 1.29, 95% CI: 1.13–1.46, FNI 5%).

**Conclusion:**

In chronic coronary syndrome, lower eGFRcys, eGFRcr, and eGFRratio were associated with higher MACE and mortality risk. eGFRcys had the strongest association; eGFRcr and eGFRratio added limited incremental value.

## Graphical abstract

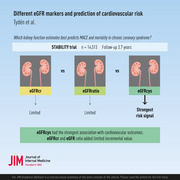

**Abbreviations**
CHF: congestive heart failure
CKD: chronic kidney disease
CV: cardiovascular
CVD: cardiovascular disease
eGFRcr: creatinine‐based eGFR
eGFRcys: cystatin C–based eGFR
FNI: fraction of new information
GFR: glomerular filtration rate
HDL: high‐density lipoprotein
KDIGO: kidney disease: improving global outcomes
LDL: low‐density lipoprotein
MACE: major adverse cardiovascular events
MCE: major coronary event
MI: myocardial infarction
PAD: peripheral artery disease
SGHS: selective glomerular hypofiltration syndrome
SPS: shrunken pore syndrome

## Introduction

Chronic kidney disease (CKD) is a major risk factor for cardiovascular disease, a leading cause of mortality among CKD patients [1, 2, 3]. Studies have shown that even in the early stages of CKD (Stages 1–3), the risk of cardiovascular (CV) events is elevated at least two‐fold compared to the general population, and the risk increases markedly as CKD progresses to more advanced stages (Stages 4–5) [4, 5]. Furthermore, in the early stages of CKD, the risk of CV death surpasses the risk of progression to advanced kidney disease [6].

Estimating glomerular filtration rate (GFR) is fundamental and plays a key role in diagnosing, staging, and managing CKD. GFR is commonly estimated using equations based on plasma or serum levels of creatinine or cystatin C. Creatinine is the most widely used biomarker, but its accuracy is limited due to interfering factors like muscle mass, sex, diet, and age [7, 8]. Kidney Disease: Improving Global Outcomes (KDIGO) guidelines recommend cystatin C as an alternative biomarker in cases where creatinine measurements are unreliable [9]. Cystatin C levels can be influenced by corticosteroid treatment, hyperthyroidism, and body composition (obesity) [10, 11, 12]. An association with inflammation has been reported in some observational studies, but not in studies in elective surgery patients with marked inflammatory response [13, 14, 15, 16, 17, 18].

When creatinine‐based eGFR (eGFRcr) and cystatin C–based eGFR (eGFRcys) are measured in the same individual, discrepancies between the two values are common, as demonstrated in previous studies [19, 20]. These intraindividual differences between eGFRcr and eGFRcys can reflect non‐GFR‐related factors, such as sarcopenia. It has also been hypothesized that a discrepancy between eGFRcr and eGFRcys reflects a selective glomerular hypofiltration; for example, that shrunken or elongated pores in the glomerular basement membrane led to reduced filtration of 5–30 kDa proteins, such as cystatin C but not creatinine (<1 kDa), contributing to observed associations with clinical outcomes. This hypothesis has been named shrunken pore syndrome or, more recently, selective glomerular hypofiltration syndrome (SGHS) [21, 22, 23, 24, 25]. Previous studies have shown that patients with an eGFRcys lower than eGFRcr experience worse clinical outcomes, including higher risks of CV events, heart failure, loss of kidney function, and mortality [26, 27, 28, 29, 30, 31]. In the first study to demonstrate an association between eGFRratio (eGFRcys/eGFRcr) and mortality, an arbitrary cutoff of 0.6 was set [32]. Studies have also reported increased mortality risk with an eGFRratio of 0.7 and consequently demonstrated that the association between eGFRratio and mortality is continuous, without a definitive threshold to define increased risk [27, 32, 33]. According to Almén et al., the increased mortality could be associated with the accumulation of proteins that drive atherosclerosis [34]. Interestingly, a recent study by McCoy et al., eGFRdifference (eGFRdiff; eGFRcys–eGFRcr) showed only a weak association with reduced clearance of middle‐sized molecules, such as β_2_‐microglobulin and β_2_‐trace protein, suggesting that impaired filtration of these solutes may not fully explain the observed link between eGFR discordance and adverse outcomes [35]. Today, there are no interventional studies demonstrating that reversal of the SGHS would lead to improved patient outcomes [24].

The aim of this study was to evaluate the association between eGFRcys, eGFRcr, and their ratio (eGFRratio) with clinical outcomes, including major adverse CV events (major adverse cardiovascular events [MACE]) and all‐cause mortality, in patients with chronic coronary syndrome. Additionally, the study compared the prognostic value of the eGFRratio to that of its individual components, eGFRcys and eGFRcr.

## Methods

### Study population

The Stabilization of Atherosclerotic Plaque by Initiation of Darapladib Therapy (STABILITY) trial included 15,828 subjects from 39 countries with chronic coronary syndrome. The study design and results have previously been published [36]. In brief, participants were randomized to receive darapladib—an inhibitor of lipoprotein‐associated phospholipase A2 intended to prevent atherosclerotic plaque development in coronary arteries—or placebo along with optimal medical therapy and were followed for a mean of 3.7 years. In this STABILITY biomarker substudy, data from 14,513 patients were analyzed to investigate the association between eGFRcys, eGFRcr, and the eGFRratio with clinical outcomes. The study was conducted in accordance with the ethical principles of the Declaration of Helsinki, the International Conference of Harmonization/Good Clinical Practice guidelines, and local regulatory requirements. Written informed consent was obtained from all patients. The trial, including the biomarker substudy, was approved by the local ethics committees.

### Outcomes

The composite primary endpoint, MACE, was defined as CV death, myocardial infarction (MI), or stroke. Secondary endpoints comprised the individual components of the primary endpoint, as well as hospitalization for heart failure and major coronary event (MCE), defined as death from coronary heart disease, MI, or urgent coronary revascularization for myocardial ischemia; and total coronary events (death from coronary heart disease, MI, hospitalization for unstable angina, or any coronary revascularization). All endpoints were centrally adjudicated [36].

### Biochemical methods

Blood samples were drawn at inclusion. Plasma cystatin C analyses were performed using turbidimetry immunoassay (Tina‐quant Cystatin C Gen.2, COBAS, Roche Diagnostics, Rotkreuz, Switzerland) at Uppsala Clinical Research Centre. Serum creatinine analyses were performed with a standardized method at QUEST laboratories. For the creatinine‐ and cystatin C–based estimations of GFR, the CKD‐EPI 2021 equations were applied [37].

### Statistical methods

Follow‐up time was defined as the number of days from randomization to the occurrence of the respective event or until censorship at the study end or death from causes other than the event studied. Cox regression models were employed to estimate unadjusted and adjusted associations between the exposure—eGFRcys, eGFRcr, eGFRratio (the adjusted models included randomized treatment, country, age, sex, body mass index, smoking, hypertension, diabetes, MI, congestive heart failure, peripheral artery disease, stroke, low‐density lipoprotein [LDL], and high‐density lipoprotein [HDL])—and each outcome. To account for potential non‐linear associations, exposures are represented as restricted cubic splines with four knots at the 5th, 35th, 65th, and 95th percentiles [38]. As non‐linearity is assumed, the full association cannot be summarized by a single value. Therefore, a relative hazard is presented for a comparison between two arbitrarily selected exposure values of eGFRratio (0.7 vs. 1.0). Any two values could have been selected, but to align with previous studies on SGHS, which have used an eGFRratio ≤ 0.7 as a cutoff to define the syndrome, 0.7 was selected as one of the values and 1.0 as a natural reference representing equality of the methods [27, 31]. Non‐linearity tests (i.e., testing the null hypothesis that the association between log relative hazard and exposure is linear) were conducted in all models.

The discriminatory ability of the models was assessed using Harrell's *C*‐index [38]. The fraction of new information (FNI) provided by the exposure in each model was calculated as one minus the adequacy index, which is one minus the ratio of the likelihood ratio statistics for the model without the exposure to the model with the exposure. The FNI can be interpreted as the proportion of information that would be lost if the exposure were removed from the model [38]. All analyses were performed using R version 4.4.3, specifically utilizing the “rms” package [39].

## Results

### Descriptive statistics

Baseline characteristics of the 14,513 patients with both eGFR methods available are presented in Table 1. The median age was 65 years, and 82% were men. Median eGFRcys was 77 (interquartile range [IQR] 61–94) and eGFRcr 79 (IQR 65–91) mL/min/1.73 m^2^.

**Table 1:** Baseline characteristics for all patients with both eGFR methods available.

Variable	Combined
	*N* = 14,513
Age (years)	65.0 (59.0–71.0)
Sex: male	81.5% (11,829)
Height (cm)	170.0 (164.0–176.0) [34]
Weight (kg)	82.7 (72.0–94.3) [23]
BMI (kg/m^2^)	28.4 (25.6–31.8) [34]
Current smoker	18.1% (2629) [1]
Prior CHF	21.4% (3102)
Hypertension	71.6% (10,394)
Diabetes	38.7% (5617)
Prior stroke	8.6% (1247)
Prior PAD	8.6% (1252)
Prior PVD	15.2% (2211)
Prior MI	59.2% (8593)
Cystatin C (mg/L)	1.0 (0.9–1.2)
Creatinine (mg/dL)	1.0 (0.9–1.2)
eGFRcys (mL/min/1.73 m^2^)	77.0 (60.8–94.0)
eGFRcrea (mL/min/1.73 m^2^)	78.6 (65.3–91.4)
Diff eGFRcysC–eGFRcrea	−0.6 (−10.6 to 9.8)
Ratio eGFRcysC/eGFRcrea	1.0 (0.9–1.1)

*Note*: *M* (a–b) represents median (Q1–Q3). *P*%(*n*) represents percentage (frequency). [*M*] represents the number of missing. Tables: risk prediction for cardiovascular outcomes‐comparing creatinine‐ and cystatin C–based glomerular filtration rate and their ratio in a large cohort of patients with chronic coronary disease: a STABILITY substudy.

Abbreviations: CHF, congestive heart failure; MI, myocardial infarction; PAD, peripheral artery disease; PVD, peripheral vascular disease.

### Primary endpoint

During the 3.7 years of follow‐up, 1499 patients experienced MACE (Table 2). Lower eGFRcys was associated with a higher risk of MACE (unadjusted hazard ratio [HR] for 60 vs. 90 mL/min/1.73 m^2^: 1.58, 95% CI 1.36–1.82). After adjustment for eGFRcr, the FNI provided by eGFRcys was 54%, and eGFRcys remained a significant predictor (HR 1.77, 95% CI 1.49–2.09). When adjusted for clinical factors, the association was attenuated but remained significant (HR 1.49, 95% CI 1.28–1.73), with improved model discrimination (*C*‐index 0.65 vs. 0.63) and FNI of 32% (Table 3). Conversely, the association between eGFRcr for 60 versus 90 mL/min/1.73 m^2^ and MACE was weaker (unadjusted HR 1.43, 95% CI 1.24–1.65). After adjusting for eGFRcys, there was no positive association (HR 0.82, 95% CI 0.68–0.97, FNI 3%). When adjusted for clinical factors, the association between eGFRcr and MACE was attenuated (HR 1.29, 95% CI 1.11–1.50), with modest incremental information (FNI 15%) (Table 4).

**Table 2:** Event rates.

Event	No. at risk	No. of events	Person‐years	Incidence rate^a^	95% CI	Follow‐up
MACE	14,513	1449	49,666	2.92	2.77–3.07	3.74
MCE	14,513	1399	49,583	2.82	2.68–2.97	3.74
Death	14,513	1063	51,239	2.07	1.95–2.20	3.74
CV death	14,513	661	51,173	1.29	1.20–1.39	3.74
Hosp HF	14,513	319	50,706	0.63	0.56–0.70	3.73
MI	14,513	698	50,037	1.39	1.29–1.50	3.74
Stroke	14,513	285	50,776	0.56	0.50–0.63	3.73

Abbreviations: CV death, cardiovascular death; Death, all‐cause death; Hosp HF, hospitalization for heart failure; MACE, major adverse cardiovascular events; MCE, major coronary events; MI, myocardial infarction.

^a^ Per 100 person years.

**Table 3:** Comparison of eGFRcys 60 versus 90 mL/min/1.73 m^2^.

Model	HR [95% CI]	*p*	*C*‐index [95% CI]	*p* (nonlin)	*C* ref	FNI
**MACE**						
Unadj	1.58 [1.36–1.82]	<0.001	0.61 [0.60–0.63]	<0.001	–	–
Adj eGFRcr	1.77 [1.49–2.01]	<0.001	0.61 [0.60–0.63]	<0.001	0.57	0.54
Adj clin	1.49 [1.28–1.73]	<0.001	0.65 [0.64–0.67]	<0.001	0.63	0.32
**MCE**						
Unajd	1.55 [1.34–1.78]	<0.001	0.59 [0.58–0.61]	<0.001	–	–
Adj eGFRcr	1.77 [1.50–2.09]	<0.001	0.60 [0.58–0.61]	<0.001	0.56	0.59
Adj clin	1.52 [1.31–1.76]	<0.001	0.64 [0.62–0.65]	<0.001	0.61	0.34
**Death**						
Unadj	2.28 [1.90–2.72]	<0.001	0.66 [0.65–0.68]	<0.001	–	–
Adj eGFRcr	2.71 [2.20–3.33]	<0.001	0.67 [0.65–0.68]	<0.001	0.61	0.57
Adj clin	1.92 [1.59–2.31]	<0.001	0.71 [0.70–0.73]	<0.001	0.69	0.28
**CV death**						
Unadj	2.37 [1.89–2.98]	<0.001	0.67 [0.65–0.69]	<0.001	–	–
Adj eGFRcr	2.86 [2.19–3.73]	<0.001	0.68 [0.65–0.70]	<0.001	0.61	0.58
Adj clin	2.12 [1.67–2.69]	<0.001	0.74 [0.72–0.76]	0.002	0.70	0.30
**Hosp HF**						
Unadj	2.95 [2.06–4.22]	<0.001	0.74 [0.71–0.77]	0.238	–	–
Adj eGFRcr	3.79 [2.51–5.73]	<0.001	0.74 [0.71–0.77]	0.401	0.66	0.51
Adj clin	2.39 [1.64–3.47]	<0.001	0.80 [0.78–0.82]	0.431	0.75	0.32
**MI**						
Unadj	1.33 [1.09–1.63]	<0.001	0.57 [0.55–0.60]	<0.001	–	–
Adj eGFRcr	1.42 [1.12–1.79]	<0.001	0.58 [0.55–0.60]	0.001	0.55	0.50
Adj clin	1.31 [1.07–1.62]	<0.001	0.62 [0.60–0.64]	<0.001	0.60	0.30
**Stroke**						
Unadj	1.42 [1.02–1.98]	<0.001	0.62 [0.58–0.65]	0.337	–	–
Adj eGFRcr	1.60 [1.09–2.33]	<0.001	0.62 [0.59–0.65]	0.487	0.57	0.48
Adj clin	1.25 [0.88–1.77]	<0.001	0.66 [0.62–0.69]	0.348	0.64	0.23

*Note*: The hazard ratios correspond to a comparison of eGFRcys 60 versus 90 mL/min/1.73 m^2^. *p* is the *p* value for overall contribution of eGFRcys in the respective model. The *C*‐index is for the specified model, that is, including all variables in the respective model. The “*C* ref” is the *C*‐index for the corresponding model without the eGFRcys and “FNI” is the fraction of new information provided by eGFRcys. Unadj: No adjustment; Adj eGFRcr: adjusted for eGFRcreatinine; MACE: major adverse cardiovascular events; MCE: major coronary events; Death: all‐cause death; CV death: cardiovascular death; Hosp HF: hospitalization for heart failure; MI: myocardial infarction; Adj clin: adjusted for randomised treatment, country, age, sex, body mass index (BMI), smoking, hypertension, diabetes, MI, congestive heart failure (CHF), peripheral artery disease (PAD), stroke, low‐density lipoprotein (LDL), and high‐density lipoprotein (HDL).

**Table 4:** Comparison of eGFRcr 60 versus 90 mL/min/1.73 m^2^.

Model	HR [95% CI]	*p*	*C*‐index [95% CI]	*p* (nonlin)	*C* ref	FNI
**MACE**						
Unadj	1.43 [1.24–1.65]	<0.001	0.57 [0.56–0.59]	<0.001	–	–
Adj eGFRcys	0.82 [0.68–0.97]	0.026	0.61 [0.60–0.63]	0.986	0.61	0.03
Adj clin	1.29 [1.11–1.50]	<0.001	0.64 [0.62–0.65]	<0.001	0.63	0.15
**MCE**						
Unadj	1.35 [1.17–1.56]	<0.001	0.56 [0.54–0.57]	<0.001	–	–
Adj eGFRcys	0.80 [0.67–0.96]	0.005	0.60 [0.58–0.61]	0.912	0.59	0.06
Adj clin	1.27 [1.09–1.48]	<0.001	0.62 [0.61–0.64]	<0.001	0.61	0.15
**Death**						
Unadj	1.71 [1.44–2.03]	<0.001	0.61 [0.59–0.62]	0.002	–	–
Adj eGFRcys	0.72 [0.58–0.88]	0.001	0.67 [0.65–0.68]	0.898	0.66	0.04
Adj clin	1.37 [1.14–1.64]	<0.001	0.69 [0.68–0.71]	<0.001	0.69	0.10
**CV death**						
Unadj	1.86 [1.50–2.32]	<0.001	0.61 [0.58–0.63]	0.002	–	–
Adj eGFRcys	0.73 [0.56–0.95]	0.002	0.68 [0.65–0.70]	0.810	0.67	0.05
Adj clin	1.56 [1.24–1.96]	<0.001	0.71 [0.69–0.73]	0.002	0.70	0.12
**Hosp HF**						
Unadj	2.07 [1.50–2.86]	<0.001	0.66 [0.63–0.69]	0.016	–	–
Adj eGFRcys	0.64 [0.44–0.94]	0.074	0.74 [0.71–0.77]	0.560	0.74	0.03
Adj clin	1.48 [1.05–2.08]	<0.001	0.77 [0.75–0.80]	0.018	0.75	0.14
**MI**						
Unadj	1.29 [1.05–1.59]	<0.001	0.55 [0.53–0.57]	0.012	–	–
Adj eGFRcys	0.91 [0.70–1.17]	0.668	0.58 [0.55–0.60]	0.973	0.57	0.02
Adj clin	1.25 [1.00–1.55]	<0.001	0.61 [0.59–0.63]	0.060	0.60	0.16
**Stroke**						
Unadj	1.23 [0.89–1.71]	<0.001	0.57 [0.54–0.61]	0.142	–	–
Adj eGFRcys	0.75 [0.51–1.11]	0.503	0.62 [0.59–0.65]	0.441	0.62	0.05
Adj clin	1.03 [0.73–1.45]	0.022	0.65 [0.62–0.68]	0.173	0.64	0.10

*Note*: The hazard ratios correspond to a comparison of eGFRcr 60 versus 90 mL/min/1.73 m^2^. *p* is the *p* value for overall contribution of eGFRcr in the respective model. The *C*‐index is for the specified model, that is, including all variables in the respective model. The “*C* ref” is the *C*‐index for the corresponding model without the eGFRcr and “FNI” is the fraction of new information provided by eGFRcr. Unadjusted: no adjustment; Adj eGFRcys: adjusted for cystatin C; MACE: major adverse cardiovascular events; MCE: major coronary events; Death: all‐cause death; CV death: cardiovascular death; Hosp HF: hospitalization for heart failure; MI: myocardial infarction; Adj clin: adjusted for randomised treatment, country, age, sex, body mass index (BMI), smoking, hypertension, diabetes, MI, congestive heart failure (CHF), peripheral artery disease (PAD), stroke, low‐density lipoprotein (LDL), and high‐density lipoprotein (HDL).

**Fig. 1:**
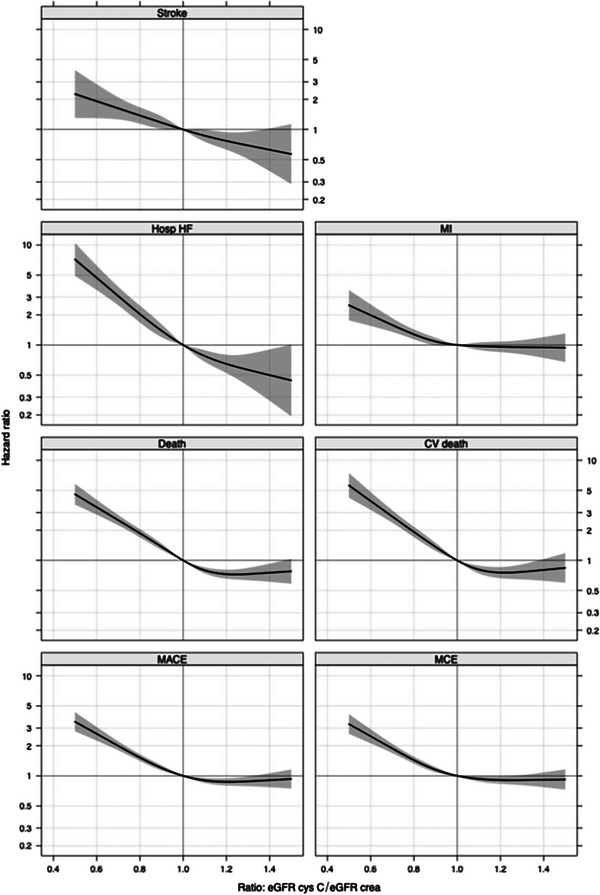
Unadjusted relative hazard of outcomes across a range of eGFR ratios, compared with the reference eGFRratio of 1.0. Risk prediction for cardiovascular outcomes‐comparing creatinine‐ and cystatin C‐based glomerular filtration rate and their ratio in a large cohort of patients with chronic coronary disease: a STABILITY substudy. CV death, cardiovascular death; Death, all‐cause death; Hosp HF, hospitalization for heart failure; MACE, major adverse cardiovascular events; MCE, major coronary events; MI, myocardial infarction.

The unadjusted continuous association between eGFRratio and the risk of MACE showed that the risk of MACE increases progressively as the eGFRratio falls below 1 (Fig. 1). Comparing an eGFRratio of 0.7 versus 1.0, the unadjusted HR was 1.99 (95% CI 1.80–2.21). After adjusting for eGFRcr, the association remained significant (HR 1.89, 95% CI 1.70–2.10) but was attenuated after adjustment for eGFRcys (HR 1.29, 95% CI 1.13–1.46) and even more attenuated after adjustment for both eGFRcr and eGFRcys (HR 1.09, 95% CI 0.58–2.04). The added value of eGFRratio measured by FNI ranged from 3% to 54%, with low percentages (FNI 1–5), observed in the model adjusted for eGFRcys and both eGFR equations. In the model adjusted for clinical factors, the association remained strong (HR 1.78, 95% CI 1.59–1.98), with improved discrimination (*C*‐index 0.66 vs. 0.65) and 19% incremental information (Table 5). Figure 2a–b illustrates the stronger association of eGFRratio with MACE when adjusted for eGFRcr compared to eGFRcys.

**Table 5:** Models for the ratio eGFRcys/eGFRcr.

Model	HR [95% CI]	*p*	*C*‐index [95% CI]	*p* (nonlin)	*C* ref	FNI
**MACE**						
Unadj	1.99 [1.80–2.21]	<0.001	0.59 [0.57–0.60]	<0.001	–	–
Adj eGFRcr	1.89 [1.70–2.10]	<0.001	0.62 [0.60–0.63]	<0.001	0.57	0.54
Adj eGFRcys	1.29 [1.13–1.46]	0.002	0.61 [0.60–0.63]	0.007	0.61	0.05
Adj eGFRcrea+cys	1.09 [0.58–2.04]	0.030	0.62 [0.60–0.63]	0.254	0.61	0.03
Adj clin	1.78 [1.59–1.98]	<0.001	0.66 [0.65–0.68]	<0.001	0.65	0.19
**MCE**						
Unadj	1.88 [1.69–2.09]	<0.001	0.58 [0.56–0.59]	<0.001	–	–
Adj eGFRcr	1.80 [1.62–2.00]	<0.001	0.60 [0.58–0.61]	<0.001	0.56	0.60
Adj eGFRcys	1.31 [1.14–1.50]	0.001	0.60 [0.58–0.61]	0.027	0.59	0.08
Adj eGFRcrea + cys	1.25 [0.65–2.42]	0.090	0.60 [0.58–0.61]	0.161	0.60	0.03
Adj clin	1.71 [1.53–1.91]	<0.001	0.66 [0.64–0.67]	<0.001	0.64	0.19
**Death**						
Unadj	2.52 [2.25–2.84]	<0.001	0.63 [0.62–0.65]	<0.001	–	–
Adj eGFRcr	2.35 [2.08–2.64]	<0.001	0.67 [0.65–0.69]	<0.001	0.61	0.57
Adj eGFRcys	1.39 [1.20–1.61]	<0.001	0.67 [0.65–0.69]	0.012	0.66	0.05
Adj eGFRcrea + cys	1.52 [0.75–3.09]	0.090	0.67 [0.65–0.69]	0.060	0.67	0.01
Adj clin	2.04 [1.80–2.32]	<0.001	0.73 [0.71–0.75]	<0.001	0.71	0.17
**CV death**						
Unadj	2.75 [2.38–3.17]	<0.001	0.64 [0.62–0.66]	<0.001	–	–
Adj eGFRcr	2.55 [2.21–2.95]	<0.001	0.68 [0.66–0.70]	<0.001	0.61	0.59
Adj eGFRcys	1.48 [1.24–1.77]	<0.001	0.68 [0.66–0.70]	0.006	0.67	0.06
Adj eGFRcrea + cys	1.45 [0.63–3.35]	0.041	0.68 [0.66–0.70]	0.100	0.68	0.02
Adj clin	2.12 [1.90–2.36]	<0.001	0.76 [0.74–0.78]	<0.001	0.75	0.14
**Hosp HF**						
Unadj	3.08 [2.49–3.82]	<0.001	0.68 [0.65–0.71]	0.042	–	–
Adj eGFRcr	2.74 [2.21–3.40]	<0.001	0.74 [0.71–0.77]	0.187	0.66	0.50
Adj eGFRcys	1.28 [0.99–1.65]	0.136	0.74 [0.71–0.77]	0.562	0.74	0.02
Adj eGFRcrea + cys	1.48 [0.47–4.63]	0.654	0.74 [0.71–0.77]	0.451	0.74	0.01
Adj clin	2.34 [1.97–2.78]	<0.001	0.83 [0.81–0.85]	<0.001	0.80	0.16
**MI**						
Unadj	1.58 [1.35–1.85]	<0.001	0.55 [0.53–0.57]	0.005	–	–
Adj eGFRcr	1.52 [1.30–1.79]	<0.001	0.58 [0.55–0.60]	0.021	0.55	0.48
Adj eGFRcys	1.13 [0.93–1.38]	0.612	0.58 [0.55–0.60]	0.563	0.57	0.03
Adj eGFRcrea + cys	0.59 [0.22–1.56]	0.409	0.58 [0.55–0.60]	0.780	0.58	0.04
Adj clin	1.56 [1.32–1.84]	<0.001	0.66 [0.64–0.68]	0.015	0.64	0.12
**Stroke**						
Unadj	1.63 [1.26–2.10]	<0.001	0.59 [0.55–0.62]	0.864	–	–
Adj eGFRcr	1.53 [1.18–1.97]	<0.001	0.62 [0.59–0.65]	0.981	0.57	0.47
Adj eGFRcys	1.04 [0.77–1.42]	0.838	0.62 [0.58–0.65]	0.902	0.62	0.02
Adj eGFRcrea + cys	0.55 [0.10–3.00]	0.858	0.62 [0.59–0.65]	0.683	0.62	0.02
Adj clin	1.55 [1.29–1.86]	<0.001	0.70 [0.67–0.73]	<0.001	0.69	0.11

*Note*: The hazard ratios correspond to a comparison between a ratio of 0.7 and a ratio of 1. *p* is the *p* value for the ratio's overall contribution in the respective model. The *C*‐index is for the specified model, that is, including all variables in the respective model. The “*C* ref” is the *C*‐index for the corresponding model without the ratio and “FNI” is the fraction of new information provided by the ratio. Unadjusted: no adjustment; Adj eGFRcr: Adjusted for eGFRcreatinine; Adj eGFRcys: adjusted for eGFR cystatin C; Adj eGFRcr + cys: adjusted for both eGFRcreatinine and eGFRcystatin C; MACE: major adverse cardiovascular events; MCE: major coronary events; Death: all‐cause death; CV death: cardiovascular death; Hosp HF: hospitalization for heart failure; MI: myocardial infarction; Adj clin: adjusted for randomised treatment, country, age, sex, body mass index (BMI), smoking, hypertension, diabetes, MI, congestive heart failure (CHF), peripheral artery disease (PAD), stroke, low‐density lipoprotein (LDL), and high‐density lipoprotein (HDL).

Fig. 2(a and b) One‐year risk for the different outcomes as a function of eGFR ratio for different eGFR levels. eGFR methods are adjusted for creatinine (a) and cystatin C (b). CV death, cardiovascular death; Death, all‐cause death; Hosp HF, hospitalization for heart failure; MACE, major adverse cardiovascular events; MCE, major coronary events; MI, myocardial infarction.

**Figure:**
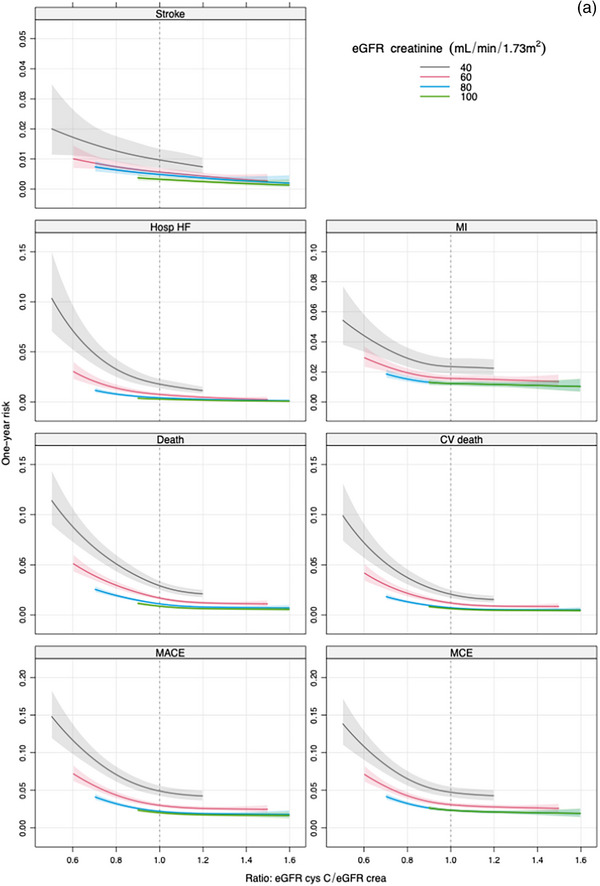

**Figure:**
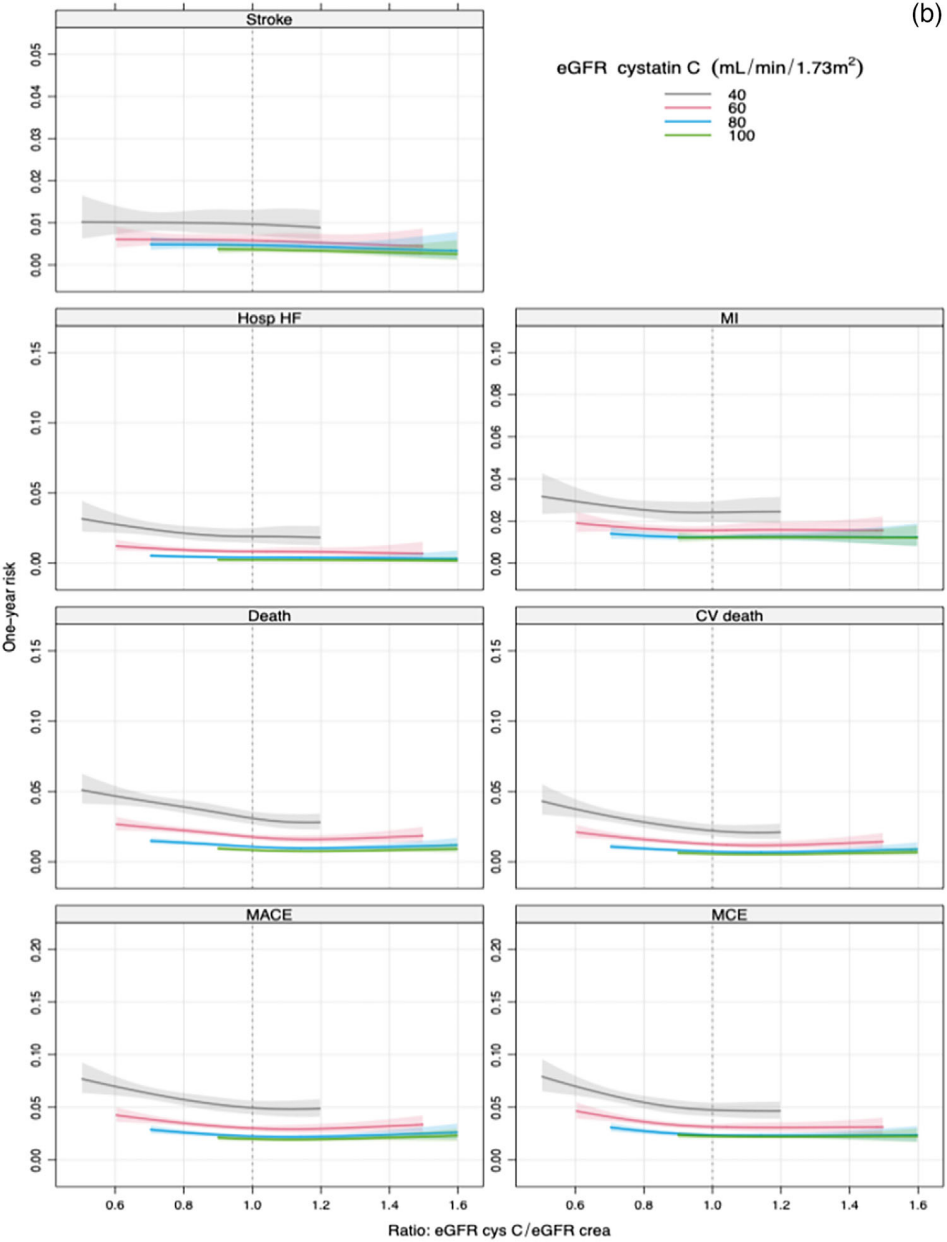

### Secondary endpoints

Throughout the study period, there were 1399 MCE, 1063 deaths from all causes, 661 deaths due to CV causes, 319 hospitalizations for heart failure, 698 MI, and 285 strokes (Table 2). Lower eGFRcys was significantly associated with increased risks for all secondary endpoints, with HRs comparing 60 versus 90 mL/min/1.73 m^2^ ranging from 1.33 (MI) to 2.95 (heart failure hospitalization) (all *p* < 0.001). In the models adjusted for eGFRcr or clinical factors, eGFRcys remained significantly associated with all secondary endpoints (Table 3). For eGFRcr (60 vs. 90 mL/min/1.73 m^2^), the unadjusted HRs for secondary endpoints ranged from 1.23 (stroke) to 2.07 (heart failure hospitalization) (all *p* < 0.001). However, after adjusting for eGFRcys, associations with heart failure hospitalization (*p* = 0.07), MI (*p* = 0.67), and stroke (*p* = 0.50) were no longer significant. After adjustment for clinical factors, eGFRcr remained significantly associated with MCE, all‐cause death, CV death, hospitalization for heart failure, and MI, although with modest incremental information (FNI 10%–16%) (Table 4).

Figure 1 illustrates the unadjusted continuous association between eGFRratio and secondary outcomes. When adjusting for eGFRcys, the associations between the eGFRratio and the secondary endpoints were attenuated. After adjustment for clinical factors, eGFRratio remained significantly associated with all secondary endpoints, though with attenuated HRs and modest incremental predictive value (Table 5). The FNI values ranged between 1.8% and 7.7% in the models adjusted for eGFRcys and 1.4%–3% when adjusted for both eGFR methods, indicating only marginal additional predictive value for eGFRratio. Figure 2a–b illustrates stronger associations with eGFRratio and secondary outcomes, when adjusted for eGFRcr compared to eGFRcys.

## Discussion

Lower eGFRcys was significantly associated with higher risk of all CV outcomes and death, whereas eGFRcr showed weaker associations that disappeared after adjustment for eGFRcys. Including eGFRcys improved prognostic value, particularly for heart failure and death. The prognostic value of eGFRcys was substantial—removing it from a model containing eGFRcr resulted in a 50%–60% loss of information for all CV outcomes. In contrast, removing eGFRcr from a model, including eGFRcys, led to <6% of information loss, indicating limited added value. Although eGFRcr remains useful for assessing kidney function, its role in CV risk prediction appears limited in the presence of eGFRcys, reinforcing what has been demonstrated in prior studies [40, 41, 42]. Apart from creatinine's susceptibility to non‐GFR determinants such as age, muscle mass, and diet, a non‐linear association between eGFRcr and CV risk has been observed [43].

In our study, a lower eGFRratio (eGFRcys/eGFRcr) was linked to an increased risk of MACE in patients with chronic coronary syndrome, and the association was continuous with no clear threshold. After adjusting for eGFRcys, the predictive value of the eGFRratio was substantially reduced for all outcomes and eGFR levels, indicating that eGFRcys is the driving factor in the eGFRratio. Comparing an eGFRratio of 0.7–1.0 revealed a significant increase in risk for all outcomes, which agrees with previous reports in different populations [26, 27, 28, 29, 30, 31]. Adjusting for eGFRcr had only a small impact on the HR for MACE, whereas adjusting for eGFRcys considerably attenuated this association.

Similar results were observed for secondary CV endpoints, with a higher risk associated with a lower eGFRratio. Adjusting for both eGFR methods diminished the predictive value of the ratio, suggesting that its additive value is limited beyond the individual eGFRcys estimate.

Contrary to some prior studies [26, 27, 28, 30, 31], our analysis highlights that although the eGFRratio is associated with CV risk, its incremental predictive value over eGFRcys or eGFRcr alone is limited. The FNI of up to 8%, provided by the eGFRratio in models adjusted for eGFRcys, reflects that the majority of prognostic information is already captured by eGFRcys.

The strong predictive power of eGFRcys is consistent with previous studies demonstrating superiority over eGFRcr in predicting all‐cause mortality and CV events [40, 41]. In a study, Hoke et al. reported that serum cystatin C, but not creatinine or eGFR (using the MDRD equation), significantly predicted MACE in patients with asymptomatic carotid atherosclerosis [44]. Similarly, Hijazi et al. reported that eGFRcys correlated more strongly than eGFRcr with CV death in patients with atrial fibrillation and that models incorporating the individual eGFR equation variables provided better discrimination than models based on the different eGFR methods [45].

Our findings confirm that a lower eGFRcys compared to eGFRcr is associated with a higher risk of CV events and mortality, supporting its role as a predictor of CV risk [42, 46, 47, 48, 49]. The stronger associations observed between eGFRcys and mortality and heart failure hospitalization may stem from its sensitivity to non‐GFR determinants like inflammation and obesity, which could influence CV disease progression. However, low molecular mass protein accumulation cannot be excluded, because these were not measured in the current study.

The prevalence of intraindividual differences in eGFR estimates has been reported to be 8%–23% in Swedish cohorts depending on the cutoff used and population studied (eGFRratio of 0.6 or 0.7) [32, 50]. Large discrepancies between eGFRcys and eGFRcr may indicate that non‐GFR‐related factors influence creatinine and cystatin C plasma levels. Conditions like sarcopenia and malnutrition lead to lower creatinine levels, resulting in overestimation of GFR. Inflammation and elevated levels of inflammatory cytokines, such as interleukin‐6 (IL‐6) or tumor necrosis factor, could raise cystatin C levels, resulting in a lower eGFRcys, even when kidney function is preserved [10, 11, 51, 52]. Notably, our knowledge on cystatin C and inflammation today relies on observed associations, and future interventional studies will contribute to increased knowledge. IL‐6 has been shown to play an important role in the inflammatory process in atherosclerosis, and elevated levels are associated with CV events [53, 54, 55]. The effect of interventions targeting IL‐6 on cystatin C levels could be investigated.

The mechanisms underlying the discordance between eGFRcys and eGFRcr remain incompletely understood [24, 33]. Structural changes in the glomerular basement membrane, as proposed in SGHS, may selectively impair filtration of medium‐sized proteins (5–30 kDa), including cystatin C, and potentially atherosclerosis‐promoting proteins [21, 25, 34]. This hypothesis provides a plausible explanation for the observed associations with adverse outcomes, but direct mechanistic evidence is still limited [23]. In line with previous observational studies [19, 56], our result shows that a lower eGFRratio is associated with CV events and mortality. The threshold defining SGHS has been established arbitrarily, and eGFRratios of 0.6–0.7 have been used in different reports [21, 26, 27, 28]. We observed a continuous risk increase without a clear threshold. Interventional studies reversing the condition are needed.

## Strength and limitations

Using data from the STABILITY trial, a large and well‐characterized cohort of patients with chronic coronary syndrome, our analysis provides a robust foundation for evaluating the prognostic utility of eGFRcys, eGFRcr, and the eGFRratio in a population with mild‐to‐moderate CKD. The study endpoints were adjudicated, and creatinine and cystatin C were measured using calibrated methods. The use of both creatinine‐based and eGFRcys equations allows for a comprehensive assessment of kidney function and its association with CV outcomes. Still, limitations must be acknowledged. The observational nature of the study precludes the establishment of causal relationships. Although we adjust for covariates, residual confounding cannot be excluded. Given the predominantly male composition of this cohort and the exclusion of more advanced stages of CKD, the generalizability might be limited. Therefore, validating these results in diverse patient populations would be valuable. Finally, the use of a single‐baseline measurement of eGFR may not fully capture the dynamic nature of kidney function over time.

## Conclusion

In patients with chronic coronary syndrome, eGFRcys was a better indicator of CV events and mortality than eGFRcr, capturing the majority of the prognostic information provided by renal dysfunction in relation to CV outcomes. The eGFRratio (eGFRcys/eGFRcr) offered only marginal additional value beyond its individual components of eGFRcr and eGFRcys. The association between eGFRratio and CV risk was continuous, with no clear thresholds, consistent with previous reports. Given its minimal incremental value, eGFRratio did not substantially improve CV risk prediction over eGFRcys alone in patients with chronic coronary syndrome.

## Author contributions

*Concept and design*: Gorav Batra, Bengt Fellström, Claes Held, Johan Lindbäck, Inga Soveri, Maria K. Svensson, Maria Tydén, and Lars Wallentin. *Statistical analysis*: Johan Lindbäck. *Analysis and interpretation of data*: Gorav Batra, Bengt Fellström, Claes Held, Johan Lindbäck, Inga Soveri, Maria K. Svensson, Ralph Stewart, Maria Tyden, Lars Wallentin and Harvey D. White. *Manuscript draft*: Maria Tydén. All authors reviewed the results and approved the final manuscript.

## Conflict of interest statement

Maria Tydén has received honoraria from AstraZeneca and GSK, not related to this manuscript. Gorav Batra reports institutional research grants from AstraZeneca, Novo Nordisk, and Pfizer, expert committee and consulting fees to his institution from Bayer. Honoraria for lectures and scientific advice from AstraZeneca, Bristol Myers Squibb, Boehringer Ingelheim, Novartis, Novo Nordisk, Pfizer, and Sanofi. Bengt Fellström has received honoraria for consulting, advisory boards, steering committees, and DMC panels from ALEXION, NOVARTIS, AstraZeneca, STADA, Calliditas, CSL Behring, BMS, but not related to the present manuscript. Claes Held reports institutional grants (not related to this manuscript) from GSK, Bayer, and Pfizer, advisory board and lecture fees from AstraZeneca, Pfizer, Boehringer Ingelheim, Novo Nordisk, Sanofi, and Amarin unrelated to the current manuscript. Johan Lindbäck has received institutional grants from GSK, not related to this manuscript. Harvey D. White has received grant support paid to the institution and fees for serving on Steering Committees from Sanofi‐Aventis, Regeneron Pharmaceuticals, Eli Lilly, Omthera Pharmaceuticals, American Regent, Eisai Inc., DalCor Pharma UK, Inc., CSL Behring, NHI, Sanofi‐Aventis Australia Pty Ltd, Esperion Therapeutics, Inc., Janssen Pharmaceuticals, and National Institutes of Health. He was on the Advisory Boards for Genentech, Inc. and VEVRE. Lars Wallentin reports no grants related to this manuscript. Inga Soveri has received honoraria from Bayer and STADA, not related to this manuscript. Maria K. Svensson has received honoraria from Amgen, AstraZeneca, Boehringer Ingelheim, GSK, and Novo Nordisk, but these are not related to this manuscript. Ralph Stewart reports no grants related to this manuscript.

## Funding information

This work was supported by Swedish State Support for Research (ALF‐agreement) and the Swedish Kidney Foundation.

## Data Availability

The data that support the findings of this study are available from the corresponding author upon reasonable request.
